# Supplementary material for: An equivariant pretrained transformer for unified 3D molecular representation learning
Source: Nat Commun. 2026 Feb 10;17:2606. doi: 10.1038/s41467-026-69185-7 (PMC13003020; doi:10.1038/s41467-026-69185-7)
Supplement: Supplementary file 1 — Supplementary Information [file 41467_2026_69185_MOESM1_ESM.pdf]

Supplementary Information: An Equivariant Pretrained Transformer for Unified 3D Molecular Representation Learning

Rui Jiao[1,2,+], Xiangzhe Kong[1,2,+], Li Zhang[1,2,+], Ziyang Yu[1,2], Fangyuan Ren[1,2,3], Wenjuan Tan[1,2], Wenbing Huang[4,5,*], and Yang Liu[1,2,*]

[1]Department of Computer Science and Technology, Tsinghua University, Beijing, China
[2]Institute for AI Industry Research, Tsinghua University, Beijing, China
[3]Department of Pharmacy, University of Pisa, Pisa, Italy
[4]Gaoling School of Artificial Intelligence, Renmin University of China, Beijing, China
[5]Beijing Key Laboratory of Research on Large Models and Intelligent Governance, Beijing, China
[*]Correspondence should be addressed to: hwenbing@ruc.edu.cn liuyang2011@tsinghua.edu.cn
[+]These authors contributed equally to this work.

List of Supplementary Notes

1 Dataset Collection **2**
1.1 Detailed Dataset Distribution . . . 2
1.2 Vocabulary Construction . . . 2
2 Comparison on Denoising Strategies **2**
3 Implementation Details **5**
3.1 Baseline Implementations on Virtual Screening Benchmark . . . 5
3.2 Hyperparameters for Pretraining . . . 5
3.3 Hyperparameters for LBA Task . . . 6
3.4 Hyperparameters for MPP Task . . . 6
3.5 Hyperparameters for MSP Task . . . 7
3.6 Hyperparameters for Virtual Screening . . . 7
4 Additional Analyses on Candidate Ligands **7**
4.1 Analysis of Outliers . . . 7
4.2 Molecular Dynamic Simulation . . . 8
4.3 Binding Free Energy Calculation . . . 9
5 Wet Lab Validation of 3CL Protease Inhibitors **11**
5.1 Compound Information . . . 11
5.2 SARS-CoV-2 3CL Protease Inhibition Assay . . . 12
6 Zero-shot Inference for RNA-Ligand Complex Affinity Prediction **12**
7 Impact of Model Scale **13**
8 Raw Results **14**
8.1 Results for LBA task . . . 14
8.2 Results for MSP task . . . 14

8.3 Results for Virtual Screening . . . 14
9 Memory Efficiency **14**
9.1 Efficient Attention Mechanism . . . 14
9.2 Comparison on Transformer-based Backbones . . . 17
10 Impact of Decoy Numbers **18**
11 Discussion on Unified Modelling for Atomic Systems **18**

List of Supplementary Figures

1 EPT rankings of marketed anti-COVID-19 drugs. . . . 8
2 A comprehensive analysis of the ten candidates screened based on EPT. . . . 10
3 Analytical data for Ac-Leu-Leu-Nle-CHO. . . . 11
4 Dose-response curve of Ac-Leu-Leu-Nle-CHO and positive control GC376 . . . 12
5 Comparison of performance of EPT and Frad under increasing layers. . . . 14
6 GPU Memory Usage for Transformer-based GNNs . . . 17
7 The effect of the number of decoys on the average rank metric during inference. . . . 18

List of Supplementary Tables

1 Statistics of the structural datasets for pretraining . . . 2
2 Construction of the vocabulary . . . 2
3 Hyperparameters for the dual-tower model . . . 5
4 Hyperparameters for constructing and training EPT . . . 6
5 Hyperparameters for finetuning on LBA . . . 6
6 Hyperparameters for finetuning on MPP . . . 6
7 Hyperparameters for finetuning on MSP . . . 7
8 Hyperparameters for finetuning on Docked-PDBBind . . . 7
9 Hyperparameters for finetuning on PDBBind non-NL complexes . . . 13
10 Results on Zero-Shot Nucleic Acid-Ligand Affinity Prediction . . . 13
11 Original runs and hyperparameter sweeps for Frad with different layers. Best results are marked in **bold**. . . . 14
12 Results on LBA . . . 15
13 Results on MSP . . . 16
14 Results on Docked PDBBind . . . 16

Supplementary Note 1. Dataset Collection

Supplementary Note 1.1. Detailed Dataset Distribution

We collection the 3D molecule datasets from small molecules and protein domain, as detailed in Supplementary Table 1. Moreover, for each time loading data from PDB, we further randomly extract a local scope with three sequencially continuous residues as a training sample. This random segmentation approach is applied as the data augmentation for proteins.

Supplementary Table 1. Statistics of the structural datasets for pretraining.

Domain	Source	# of entries	# of blocks
Small Molecule	GEOM-QM9	430,201	4,222,196
	GEOM-Drugs	1,465,181	38,222,235
	PCQM4Mv2	3,378,606	51,090,224
Protein	PDB	599,699	155,200,297
	PDBBind-PP	2,852	2,990,905
	PDBBind-refined-set	5,316	2,438,652
	PDBBind-v2020-other-PL	14,127	7,089,084

Supplementary Note 1.2. Vocabulary Construction

The vocabulary of atom types, block types and position indexes are detailed in Supplementary Table 2.

Supplementary Table 2. Construction of the vocabulary of atom types, block types and position indexes.

Vocabulary	Index	Descriptions
Atom	0∼2	<pad>, <mask>, <global>
	3∼120	118 elements
Block	0∼3	<pad>, <mask>, <unk>, <global>
	4∼23	20 amino acids
	24∼141	118 elements (H is included for completeness)
Position	0∼2	<pad>, <mask>, <global>
	3∼12	position codes for atoms in protein, i.e. α, β, etc.
	13	<sml>for atoms in small molecules

Supplementary Note 2. Comparison on Denoising Strategies

In this section, we describe the block-level denoised pretraining approach designed to incorporate the hierarchical information into our backbone model (denoted as φ hereinafter). The algorithm leverages the concept of Denoising Score Matching (DSM, [27]) to enable the model to learn from perturbed data representations. Generally, the overview of DSM is outlined in Algorithm 1. The training process begins by sampling perturbed coordinates $\mathbf{Z}'$ from a predefined noise distribution

parameterized by σ (Line 2). The atom-level pseudo forces $\mathbf{F}'$ are then predicted by the model φ to recover $\mathbf{Z}$ from $\mathbf{Z}'$ (Line 3), and finally used to compute the denoising loss $\mathcal{L}$ (Line 4). The key points of DSM lie in the design of the perturbation mechanism and the corresponding loss function to align the predicted forces $\mathbf{F}'$ with the Denoising Force Field (DFF). In the following, we first introduce the simple atom-level denoising method, then extend the denoising targets from atoms to blocks, and finally apply additional rotations on blocks to better depict the geometric landscape.

Algorithm 1 Overview of Denoised Pretraining

1: **Input:** Original Coordinates $\mathbf{Z}$, Noise scale σ, Backbone model φ.
2: Sample perturbed coordinates $\mathbf{Z}' \sim p_\sigma(\mathbf{Z}'|\mathbf{Z})$.
3: Predict pseudo forces $\mathbf{F}' \leftarrow \varphi(\mathbf{Z}')$.
4: Acquire denoising loss $\mathcal{L}(\mathbf{F}', \mathbf{Z}', \mathbf{Z})$
5: Minimize $\mathcal{L}$

Atom-level Denoising. The atom-level denoising process [10, 37] independently introduces Gaussian noise to each atom's coordinate as $\epsilon_{\mathbf{Z}} \sim \mathcal{N}(0, \mathbf{I}_{3N})$ rescaled by σ_t:

$$\mathbf{Z}' = C(\mathbf{Z} + \sigma_t \epsilon_{\mathbf{Z}}), \tag{25}$$

where the operation $C(\mathbf{Z}) = \mathbf{Z} - \sum \mathbf{Z}/N$ projects the noised sample to the mean-centered subspace to neutralize the translation introduced by the noises [35].

The training objective is to match the predicted $\mathbf{F}'$ with the denoising force field yielded by $\nabla_{\mathbf{Z}'} p_{\sigma_t}(\mathbf{Z}'|\mathbf{Z})$ as

$$\mathcal{L}_{\text{atom}} = \mathbb{E}_{\epsilon_{\mathbf{Z}} \sim \mathcal{N}(0, \mathbf{I}_{3N})} \Big[\| \mathbf{F}' - \frac{\mathbf{Z}' - \mathbf{Z}}{\sigma_t^2} \|_2^2 \Big]. \tag{26}$$

Translation-only Block-level Denoising. To conserve the intra-block geometry, we extend the atom-level denoising task into block-level by considering blocks as rigid bodies, and all the atoms within the same block are applied by the same noise. For simplification, we first define the operators $\mu_b : \mathbb{R}^{3\times N} \to \mathbb{R}^{3\times M}, g_b : \mathbb{R}^{3\times M} \to \mathbb{R}^{3\times N}$ denote the atom-to-block averaging and the block-to-atom duplication. In particular, we have

$$\begin{cases} \mu_b(\mathbf{Z})[:, m_i] = \frac{\sum_{m_j = m_i} \mathbf{z}_j}{\sum_{m_j = m_i} 1}, \\ g_b(\mathbf{Z}_b)[:, i] = \mathbf{Z}_b[:, m_i]. \end{cases} \tag{27}$$

Slightly different from the atom-level setting, we apply noises on the center of each block as $\epsilon_{\mathbf{Z}_b} \sim \mathcal{N}(0, \mathbf{I}_{3M})$. For noise scale σ_t, the perturbation and mean-centered projection are sequentially calculated as

$$\mathbf{Z}' = C\big(\mathbf{Z} + \sigma_t g_b(\epsilon_{\mathbf{Z}_b})\big). \tag{28}$$

The training objective adapts Eq. (26) into block-level as

$$\mathcal{L}_{\text{block-T}} = \mathbb{E}_{\epsilon_{\mathbf{Z}_b} \sim \mathcal{N}(0, \mathbf{I}_{3M})} \Big[\| \mu_b(\mathbf{F}') - \frac{\mu_b(\mathbf{Z}') - \mathbf{Z}_b}{\sigma_t^2} \|_2^2 \Big]. \tag{29}$$

Complete Block-level Denoising. (Ours) Simply reducing $\mathbf{F}'$ into $\mu_b(\mathbf{F}')$ in Eq. (29) ignores the torques applied on the blocks. To complete this point, we design an additional rotation denoising task from the perspective of Euler's rotation equation [28], which is previously proved effective on complex binding tasks [11].

To begin with, the torque on each block is aggregated as

$$\mathbf{M}_b'[:, m_i] = \sum_{m_j=m_i} (\mathbf{z}_j - \mathbf{Z}_b[:, m_i]) \times \mathbf{f}_j', \tag{30}$$

According to Euler's rotation equation, the time derivative of the angular momentum of each block is given by

$$\frac{d\mathbf{L}_b}{dt} = \mathbf{M}_b' = \mathbf{I}_b \alpha_b, \tag{31}$$

where $\mathbf{I}_b \in \mathbb{R}^{3\times 3\times M}$ represents the inertia matrix defined as

$$\mathbf{I}_b[:, :, m_i] = \sum_{m_j=m_i} \Big(\|\mathbf{u}_j\|^2\mathbf{I} - \mathbf{u}_j\mathbf{u}_j^\top\Big), \tag{32}$$

$$\mathbf{u}_j = \mathbf{z}_j - \mathbf{Z}_b[:, m_i]. \tag{33}$$

The angular acceleration $\alpha_b \in \mathbb{R}^{3\times M}$ can be calculated by combining Eq. (31-32) as

$$\alpha_b = \mathbf{I}_b^{-1}\mathbf{M}_b'. \tag{34}$$

To design an objective on α_b, we additionally perturb blocks by random rotations ω_b sampled from the isotropic Gaussian distribution $\mathcal{IG}_{SO(3)}(\sigma_r)$ [16]. Specifically, each rotation $\omega_b[:, m_i] \in \mathfrak{so}(3)$ is constructed as $\omega = \theta\hat{\omega}$, where $\hat{\omega}$ is a uniformly sampled unit vector, and $\theta \in [0, \pi]$ is a rotation angle with density

$$f(\theta) = \frac{1-\cos\theta}{\pi}\sum_{l=0}^{\infty}(2l+1)e^{-l(l+1)\sigma_r^2}\frac{\sin\big((l+1/2)\theta\big)}{\sin(\theta/2)}. \tag{35}$$

And the corresponding rotation matrix $\mathbf{Q}(\omega) \in SO(3)$ is acquired by the exponential mapping on $\omega = (\omega_x, \omega_y, \omega_z)^\top$:

$$\mathbf{Q}(\omega) = \exp\begin{bmatrix} 0 & -\omega_z & \omega_y \\ \omega_z & 0 & -\omega_x \\ -\omega_y & \omega_x & 0 \end{bmatrix}. \tag{36}$$

Overall, the perturbation scheme combining block-level translation and rotation is designed as

$$\mathbf{Z}_r = \mathbf{Z} - g_b(\mathbf{Z}_b), \tag{37}$$

$$\mathbf{Z}' = C\Big(g_b(\mathbf{Z}_b + \sigma_t\epsilon_{\mathbf{Z}_b}) + \mathbf{Q}_b\mathbf{Z}_r\Big). \tag{38}$$

The translation loss is defined in Eq. (29), and the rotation loss is defined as

$$\mathcal{L}_{\text{block-R}} = \mathbb{E}_{\omega\sim\mathcal{IG}_{SO(3)}(\sigma_r)}\Big[\|\alpha_b - \nabla_\omega p(\omega)\|_2^2\Big]. \tag{39}$$

The complete block-level training objective is added as

$$\mathcal{L}_{\text{block-C}} = \mathcal{L}_{\text{block-T}} + \mathcal{L}_{\text{block-R}}. \tag{40}$$

Supplementary Note 3. Implementation Details

Supplementary Note 3.1. Baseline Implementations on Virtual Screening Benchmark

We benchmark EPT with the conventional software Glide [6] and the dual-tower model ESM+Uni-Mol, which utilizes the representations of proteins and small molecules from ESM [19] and Uni-Mol [40], respectively.

Glides. We used Schrödinger Suite 2021-2 for docking. Protein structures were preprocessed with PrepWizard by adding hydrogen atoms and optimizing with the OPLS3 force field at pH 7.4. Ligands were prepared using LigPrep, preserving the original chirality, while Epik was used to predict pKa values and generate protonation states at pH 7.0. Ligand conformations were optimized with the S-OPLS force field, and a single conformation was retained for docking. The receptor grid was generated with an innerbox size of 10 × 10 × 10 and an outerbox defined as $(x_{\text{max}} - x_{\text{min}} + 20) \times (y_{\text{max}} - y_{\text{min}} + 20) \times (z_{\text{max}} - z_{\text{min}} + 20)$, with the force field set to OPLS3 and other parameters left at default values. Docking was then carried out using Glide SP (standard precision).

ESM+Uni-Mol. We used the docked-PDBBind dataset to ensure consistency across all methods. For the protein representation, we adopted the pretrained model esm2_t33_650M_UR50D[1], generating an embedding of shape (R, 1280), where R denotes the number of amino acid residues in the protein. We then computed the protein-level embedding by averaging the residue-wise embeddings. For the ligand representation, we employed the UniMolRepr function from the unimol_tools[2] package to obtain a 512-dimensional ligand embedding. To combine the two modalities, we utilized a standard dual-tower architecture. Specifically, for each protein-ligand pair, we concatenated the protein and ligand embeddings as the joint input. This concatenated vector was passed through a Feed Forward Network (FFN) with 4 hidden layers and 1 output layer, where the hidden size is 512 and the activation function is SiLU. The model was trained with a learning rate of 5e-4, and all remaining setups, including the training objective and negative rates, were kept identical to those used in our EPT model. For convenience, we denote the dimension of the concatenated representations as h_{in}. The hyperparameters for the dual tower model are listed in Supplementary Table 3.

Supplementary Table 3. Hyperparameters for the dual-tower model.

Name	lr	batch_size	epoch	h_{in}	h_{ffn}	activation
ESM+Uni-Mol	1.0×10^{-3}	16	50	1792	512	SiLU

Supplementary Note 3.2. Hyperparameters for Pretraining

We pretrain EPT on 8 NVIDIA Tesla A800 with hyperparameters in Supplementary Table 4.

[1]https://github.com/facebookresearch/esm
[2]https://unimol.readthedocs.io/en/latest

Supplementary Table 4. Hyperparameters for constructing and training EPT.

Name	h_{hidden}	h_{ffn}	h_{edge}	h_{rbf}	L	H	δ_{max}	δ_{topo}
Value	512	512	64	64	6	8	10.0	1.6
Name	epoch	scheduler	lr	min_lr	σ_t	σ_r	max_n_vertex	max_vertex_per_gpu
Value	50	cosine	1.0×10^{-4}	1.0×10^{-5}	0.04	0.1	5,000	10,000

Supplementary Note 3.3. Hyperparameters for LBA Task

We utilize the pretrained model as the encoder and additional apply an output head to predict the affinity. Specifically, we consider three types of output heads based on an MLP φ_E as follows:

$$\varphi_{\text{atom}}(\mathbf{H}^{(l)}) = \sum_i \varphi_E(\mathbf{h}_i^{(l)}), \tag{41}$$

$$\varphi_{\text{block}}(\mathbf{H}^{(l)}) = \sum_{m_i} \varphi_E\Big(\sum_{m_j=m_i} \mathbf{h}_j^{(l)})\Big), \tag{42}$$

$$\varphi_{\text{graph}}(\mathbf{H}^{(l)}) = \varphi_E(\sum_i \mathbf{h}_i^{(l)}). \tag{43}$$

Based on these heads, the hyperparameters for finetuning on LBA are provided in Supplementary Table 5, with top-k checkpoints averaged for evaluation.

Supplementary Table 5. Hyperparameters for finetuning on LBA.

Name	lr	batch_size	output_head	label_norm	epoch	save_topk	factor	patience	min_lr	omit_sml_pos
Sequence Identity 30										
EPT-Scratch	1.0×10^{-4}	16	graph	std	5	3	0.8	5	1.0×10^{-7}	True
EPT-Molecule	1.0×10^{-5}	16	block	std	15	5	0.8	5	1.0×10^{-7}	True
EPT-Protein	1.0×10^{-5}	8	graph	none	15	3	0.8	5	1.0×10^{-7}	False
EPT-MultiDomain	1.0×10^{-4}	16	graph	mad	15	5	0.8	5	1.0×10^{-7}	False
Sequence Identity 60										
EPT-Scratch	5.0×10^{-5}	8	block	none	30	5	0.8	5	1.0×10^{-7}	True
EPT-Molecule	5.0×10^{-5}	8	atom	mad	30	3	0.8	5	1.0×10^{-7}	True
EPT-Protein	5.0×10^{-5}	8	atom	none	30	5	0.8	5	1.0×10^{-7}	False
EPT-MultiDomain	5.0×10^{-5}	16	atom	none	30	5	0.8	5	1.0×10^{-7}	False

Supplementary Note 3.4. Hyperparameters for MPP Task

Following previous studies [5, 37], we utilize the noisy node technique [9] by adding $\mathcal{L}_{\text{block-C}}$ as an auxiliary training objective, and the entire loss for finetuning on QM9 can be formulated as $\mathcal{L} = \mathcal{L}_{\text{MAE}} + \lambda\mathcal{L}_{\text{block-C}}$, where λ balances the weight of each term. We utilize the same hyperparameters for all 12 tasks, which are detailed in Supplementary Table 6.

Supplementary Table 6. Hyperparameters for finetuning on MPP.

Name	lr	batch_size	σ_t	σ_r	λ	epoch	factor	patience	min_lr	omit_sml_pos
EPT	5.0×10^{-5}	64	0.04	0.1	0.1	1,000	0.8	15	1.0×10^{-7}	True
EPT-10	5.0×10^{-5}	64	0.04	0.05	0.1	1,000	0.8	15	1.0×10^{-7}	True

Supplementary Note 3.5. Hyperparameters for MSP Task

We use the split by sequence identity over 30% provided by Atom3D [30], and extract all residues within 6Å distance to the mutation point as the local view for input, where the distance between two residues is measured by the minimum distance between atom pairs. The hyperparameters for finetuning MSP are listed in Supplementary Table 7, with the last checkpoint among the top-k checkpoints for evaluation.

Supplementary Table 7. Hyperparameters for finetuning on MSP.

Name	lr	batch_size	epoch	save_topk	factor	patience	min_lr
EPT (w/o Pretrain)	1.0×10^{-5}	16	10	3	0.6	5	5.0×10^{-6}
EPT (w/ Pretrain)	1.0×10^{-5}	16	10	2	0.6	5	5.0×10^{-6}

Supplementary Note 3.6. Hyperparameters for Virtual Screening

For the ranking task, we use negative_rate to adjust the fraction of negative data pairs during training. Additionally, if local_mask is set to be true, then only attentions from neighbors within δ_{max} of each node will be calculated, otherwise attention values from all neighbors will be aggregated. The hyperparameters for finetuning Docked-PDBBind are listed in Supplementary Table 8.

Supplementary Table 8. Hyperparameters for finetuning on Docked-PDBBind, where `pred` indicates the affinity prediction task and `rank` indicates the ranking task.

Name	lr	batch_size	epoch	negative_rate	local_mask
EPT (`pred`)	3.0×10^{-5}	16	50	0.0	True
EPT (`rank`)	3.0×10^{-5}	16	50	0.6	False

Supplementary Note 4. Additional Analyses on Candidate Ligands

Supplementary Note 4.1. Analysis of Outliers

Xocova adopts a non-peptidic triazine scaffold, which is markedly different from the peptidic or peptidomimetic structures of mainstream 3CL protease inhibitors such as Leritrelvir, Rupintrivir, 3CLpro-1, GC376, Simnotrelvir, Nirmatrelvir, and Lufotrelvir, as shown in Supplementary Fig. 1. This observation suggests that although EPT exhibits strong discriminative power for mainstream structures, its performance still has room for improvement in identifying and evaluating compounds with novel scaffolds. We also notice that while most of the inhibitors are ranked at top levels, Nirmatrelvir and Lufotrelvir are underestimated as the 173-rd and 176-th. This phenomenon can be explained by our screening workflow. Target pockets and candidate ligands are first docked with the docking software, and EPT then re-scores these complexes. As a result, the performance can be constrained when truly active ligands are assigned unfavorable docking poses. Specifically, Nirmatrelvir and Lufotrelvir have Glide score ranks of 218-th and 203-rd, respectively. EPT partially corrected Glide's ordering but could not fully overcome the limitations of suboptimal docking. Further improvements in the upstream docking software are expected to enhance the final performance. Taken together, these results highlight both the strength and the current limitations of EPT, reflecting the challenges of generalizing across diverse inhibitor chemotypes.

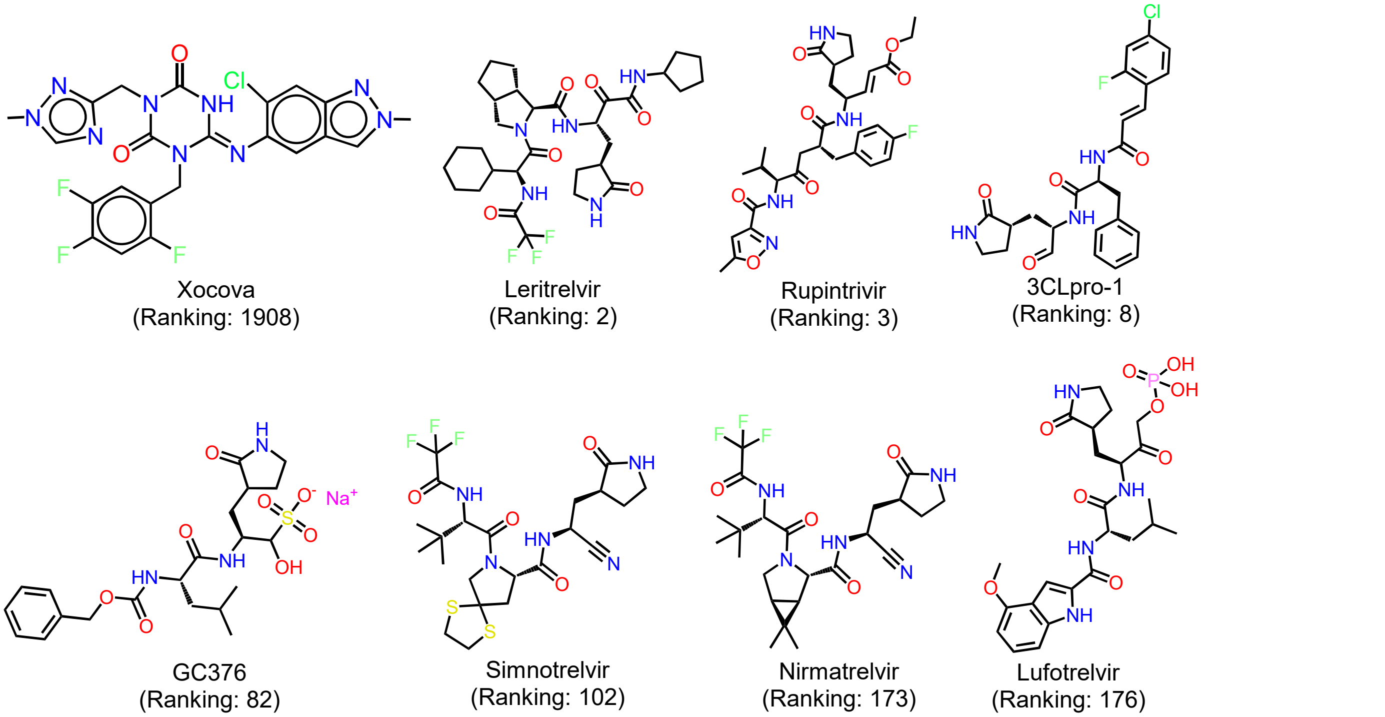

Supplementary Fig. 1 | **EPT rankings of marketed anti-COVID-19 drugs.** Global rankings of eight marketed anti-COVID-19 drugs based on EPT scores, along with their 2D chemical structures.

Supplementary Note 4.2. Molecular Dynamic Simulation

The SARS-CoV-2 3CL^{pro}-ligand complexes were simulated using GROMACS 2024.2 [1]. SARS-CoV-2 3CL^{pro} was topologized in the Amber14sb force field [24]. AmberTools 21.12 and Sobtop were used to paramterize the ligands in the general Amber force field (GAFF) [4, 21]. The Restrained Electrostatic Potential (RESP) method [32] which was calcaulated by Multiwfn [20] and quantum chemical calculation (B3LYP functional at 6-31G(d,p)) were carried out to assign the atomic charge of the ligands by using Gaussian [7].

The parameterized SARS-CoV-2 3CL^{pro}-inhibitor complex was placed in a cubic simulation box, ensuring a minimum distance of 1.0 nm between the complex and the box edges. The system was then solvated with TIP3P water molecules. Counter ions (*e.g.*, Na^+ or Cl^-) were added to neutralize the solvated system. Following solvation and ion addition, energy minimization was performed using the steepest descent algorithm to remove any unfavorable contacts. This was followed by a restrained constant number of particles, volume, and temperature (NVT) ensemble equilibration for 100 ps, and a constant number of particles, pressure, and temperature (NPT) ensemble equilibration for an additional 100 ps.

Thermodynamic properties such as pressure, density, potential energy, and temperature of the systems were monitored to ensure adequate equilibration before the production run. The Particle Mesh Ewald (PME) method was used to calculate the long-range electrostatics method with a Fourier grid spacing of 1.2 Å [14]. The LINCS algorithm [25] was applied to constrain covalent bonds during the equilibration steps. A modified V-rescale [3] thermostat and Parrinello-Rahman barostat were used for temperature and pressure coupling, respectively. Finally, 100 ns of unrestrained production simulations were carried out for the systems at 310 K and 1 bar atmospheric pressure.

Supplementary Note 4.3. Binding Free Energy Calculation

The program MMPBSA 1.6.3 [31] was developed to compute the end state-free energies of protein-ligand complexes from GROMACS MD trajectory data. Binding free energy predictions were made using an MM/PBSA approach from the MD simulation trajectories in explicit solvent, analyzing three components: the complex, receptor, and ligand [15]. In our calculation, the trajectory data from the last 10 ns was utilized, comprising 40 complex frames. The binding free energy $\Delta G_{\text{binding}}$ of the lead compounds in complex with the protein was determined using the following equation:

$$\Delta G_{\text{binding}} = \Delta G_{\text{complex}} - (\Delta G_{\text{protein}} + \Delta G_{\text{ligand}}). \tag{44}$$

In this equation, $\Delta G_{\text{complex}}$ represents the energy of the lead compound-protein complexes, and $\Delta G_{\text{protein}}$ and ΔG_{ligand} demonstrate the energy of proteins and ligands in an aqueous environment, respectively [36]. Supplementary Fig. 2 shows a comprehensive analysis of the ten candidates screened based on EPT, which also includes the $\Delta G_{\text{binding}}$ of these ten candidates.

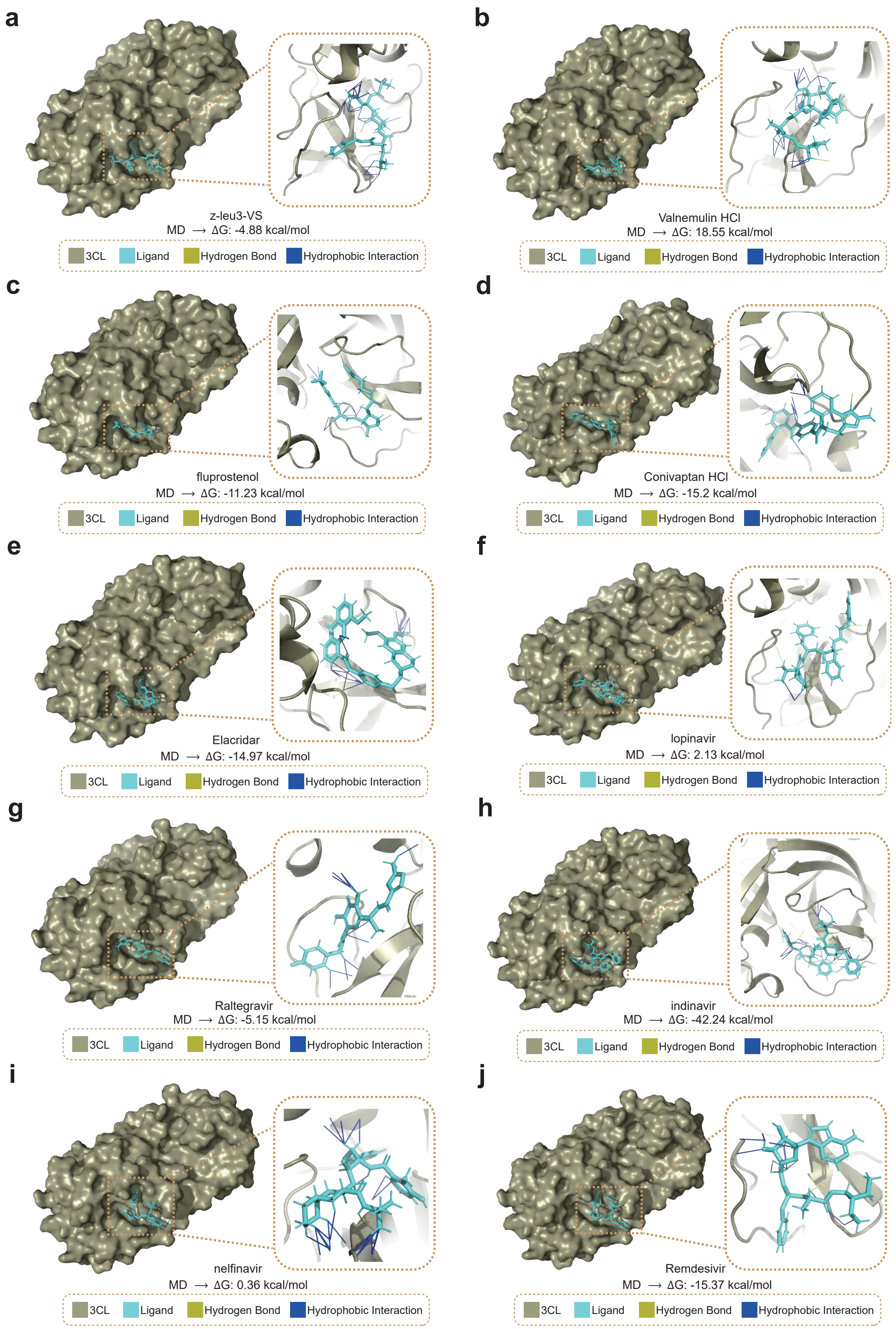

Supplementary Fig. 2 | A comprehensive analysis of the ten candidates screened based on EPT. a-e, Docking visualization and MD simulation results for the remaining molecules, excluding Ac-Leu-Leu-Nle-CHO, among the top six drugs ranked from the 1,944 FDA-approved drugs. **f-j**, Docking visualization and MD simulation results for the remaining molecules, excluding Saquinavir, among the top six drugs ranked from the 26 antiviral drugs. Note: The positive reference for the above ten candidates is also Leritrelvir ($\Delta G = -6.16\,\mathrm{kcal/mol}$).

Supplementary Note 5. Wet Lab Validation of 3CL Protease Inhibitors

Supplementary Note 5.1. Compound Information

The compound Ac-Leu-Leu-Nle-CHO (also known as MG-101; CAS No. 110044-82-1) was synthesized and purchased from MedChemExpress (MCE, Cat# HY-18964). The compound was supplied with a reported purity of ≥98% and was used without further purification. Its identity and purity were confirmed by MCE based on mass spectrometry (MS) and nuclear magnetic resonance (NMR) analysis, as shown in Supplementary Fig. 3.

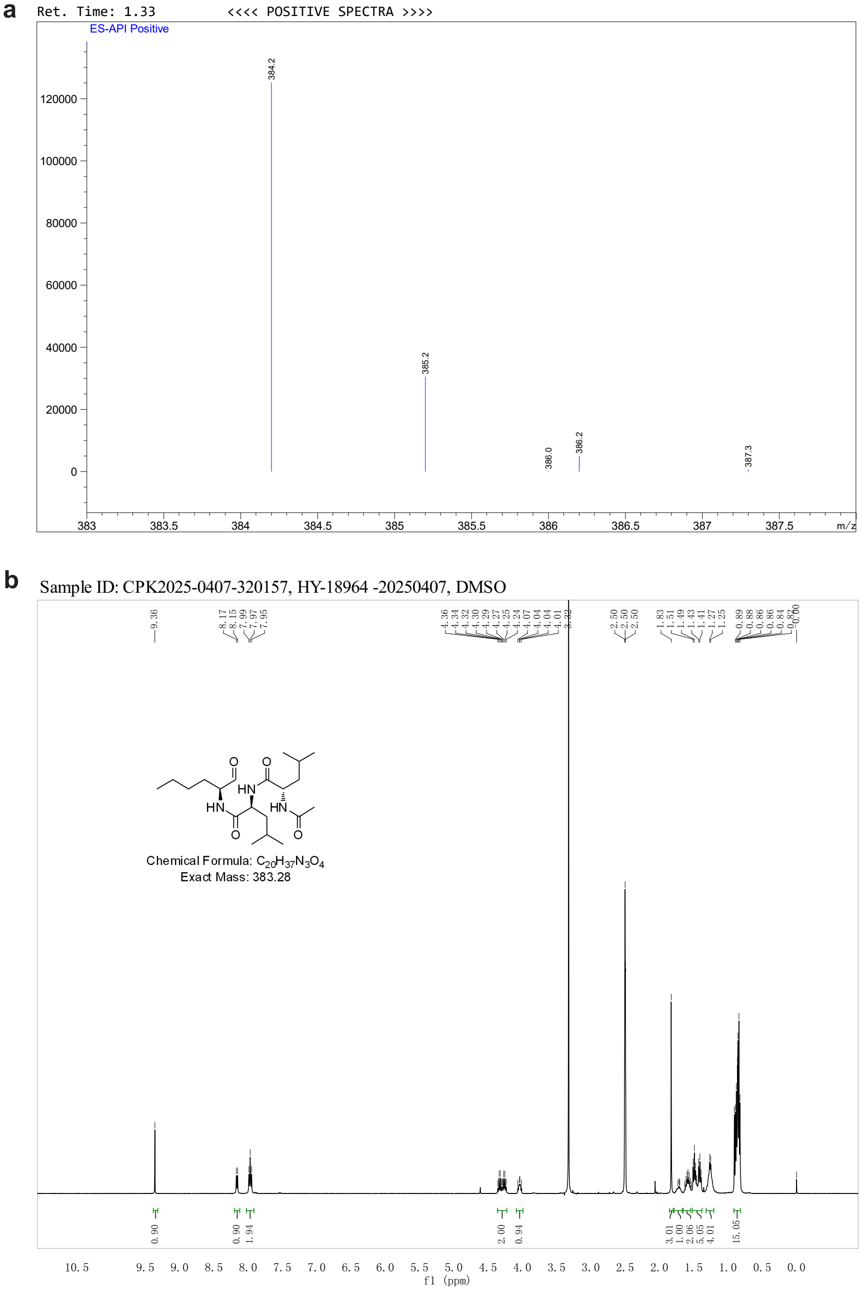

Supplementary Fig. 3 | **Analytical data for Ac-Leu-Leu-Nle-CHO. a**, ESI-MS spectrum showing the $[M+H]^+$ peak at m/z 384.2, consistent with the expected molecular weight (383.28). **b**, ^1H NMR spectrum (DMSO-d_6) confirming the chemical structure and purity of the compound. **Source**: Certificate of Analysis provided by MedChemExpress.

Supplementary Note 5.2. SARS-CoV-2 3CL Protease Inhibition Assay

The inhibitory activity of Ac-Leu-Leu-Nle-CHO against SARS-CoV-2 3CL protease was evaluated by TaoShu Bioscience (Shanghai, China). The compound was prepared as a 10 mM stock solution in DMSO and serially diluted using a 10-point, 3-fold dilution series starting from 5 mM. Diluted compound (80 nL per well, transferred by Echo) was dispensed into 384-well plates (Corning 4514), with each well adjusted to a final volume of 8 μL (top dose 50 μM, final DMSO 1%).

Recombinant SARS-CoV-2 $3CL_{pro}$ was diluted in assay buffer (50 mM HEPES pH 7.5, 2 mM DTT, 0.01% Triton X-100, 0.01% BSA) to 20 nM, and 4 μL enzyme was pre-incubated with compound at room temperature for 30 minutes. The reaction was initiated by addition of 4 μL of a fluorogenic peptide substrate (HiLyte Fluor 488-ESATLQSGLRKAK(QXL520)-NH_2, final 2 μM).

After 60 minutes at room temperature, fluorescence (Ex/Em 480/540 nm) was measured using an EnVision plate reader (PerkinElmer). Percent inhibition was calculated relative to positive and negative controls. IC_{50} values were determined by four-parameter logistic fitting (4PL, XLfit Model 205, Microsoft Excel), and dose-response curves were plotted in Python (`matplotlib`) using the fitted parameters. All experiments were performed in duplicate ($n = 2$). GC376 (positive control, provided by TaoShu Bioscience, Shanghai, China) was included for comparison under the same assay conditions. The resulting IC_{50} of 9.54 nM is comparable to the reported value of 30 nM in previous literature [23], indicating the reliability of the experimental settings.

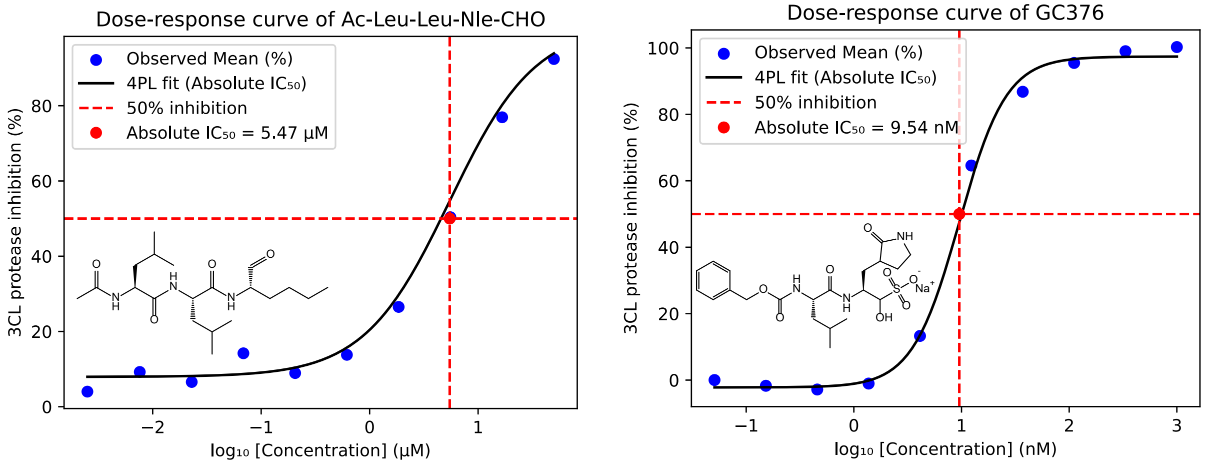

Supplementary Fig. 4 | Dose-response curves of Ac-Leu-Leu-Nle-CHO and positive control GC376. Absolute IC_{50} values were 5.47 μM for Ac-Leu-Leu-Nle-CHO and 9.54 nM for GC376, calculated using a 4PL model. Both curves were plotted in Python with fitted parameters from the experimental report. GC376 (positive control) was obtained from TaoShu Bioscience (Shanghai, China) and tested under the same assay conditions.

Supplementary Note 6. Zero-shot Inference for RNA-Ligand Complex Affinity Prediction

To demonstrate the generalization capability of EPT on biomolecules, we further extended the EPT vocabulary to nucleic acids and performed zero-shot inference on RNA-ligand complexes. Specifically, we selected Protein-Protein (PP), Protein-Nucleic acid (PN), and Protein-Ligand (PL) complexes with known Kd values from the PDBBind dataset for training. The training target is defined as $pK = -\log_{10}(\mathrm{Kd})$. Based on the pretrained model weights, we expanded the block vocabulary by adding four deoxyribonucleic acids and four ribonucleic acids, enabling EPT to

process nucleic acid data. During training, the model architecture remained consistent with the fine-tuning setup on LBA, with specific parameters provided in Supplementary Table 9.

Supplementary Table 9. Hyperparameters for finetuning on PDBBind non-NL complexes.

Name	lr	batch_size	output_head	label_norm	epoch	save_topk	factor	patience	min_lr	omit_sml_pos
EPT	1.0×10^{-5}	4	graph	none	20	5	0.8	5	1.0×10^{-7}	True

After training, we selected the latest checkpoint for evaluation and performed zero-shot inference on Nucleic acid-Ligand (NL) complexes from PDBBind. Under consistent hyperparameters, we conducted a comparison experiment between the pretrained model and that trained from scratch, with experimental results presented in Supplementary Table 10. The experiment demonstrates that pretraining provides a significant boost to the model's zero-shot performance, highlighting the strong generalization ability of EPT across different biomolecular modalities. This result underscores the potential of EPT for more diverse molecular interaction tasks.

Supplementary Table 10. The mean and standard deviations of 3 runs on the PDBBind NL complexes.

Model	Pearson↑	Spearman↑
EPT-pretrain	0.402±0.037	0.315±0.058
EPT-scratch	0.359±0.057	0.263±0.061

Supplementary Note 7. Impact of Model Scale

To investigate the scalability of EPT, we conducted controlled experiments on QM9 by scaling both EPT and a powerful pretrained model, Frad [5], from 6 to 12 layers. For layers higher than the default setting (L=8), we further applied a hyperparameter sweep on the learning rate of Frad among $\{5e-4, 4e-4, 1e-4, 5e-5, 1e-5\}$ and recorded the best results. The results are summarized in Supplementary Fig. 5. We observe that EPT exhibits consistent performance improvements as the number of layers increases from 6 to 10, with performance saturating or marginally improving at 12 layers. This indicates that EPT benefits from deeper architectures and scales robustly with increased model capacity. In contrast, Frad does not exhibit consistent gains with larger model sizes. Specifically, its performance fluctuates beyond 8 layers, which is further shown in Supplementary Table 11.

This difference suggests that EPT not only has better scalability but also offers more stable optimization behavior when scaled up. Based on this observation, we selected a 10-layer backbone (EPT-10) to fully leverage EPT's scalability while balancing computational cost.

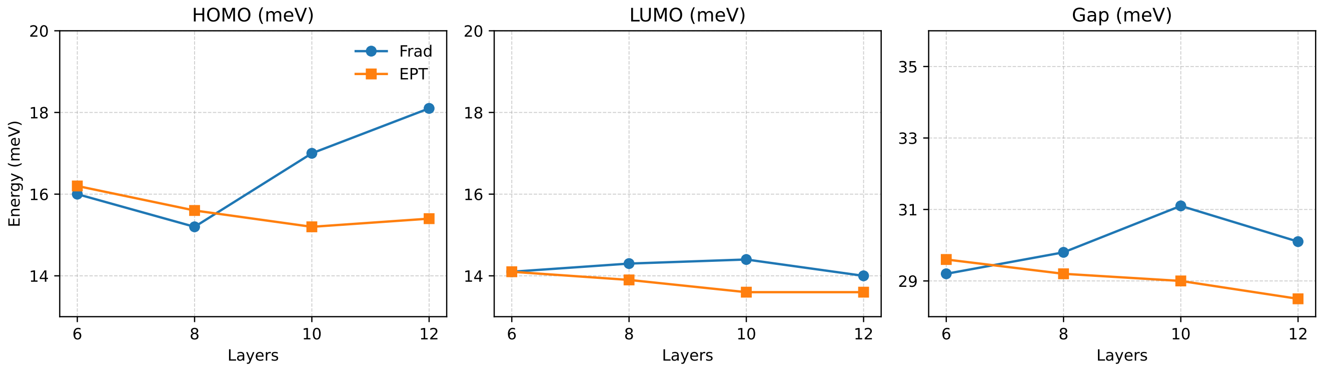

Supplementary Fig. 5 | Comparison of performance of EPT and Frad under increasing layers. Mean absolute errors (MAE) on HOMO, LUMO, and Gap prediction are reported for both EPT and Frad with varying numbers of layers (6, 8, 10, and 12). EPT demonstrates consistent performance improvements as the model depth increases. In contrast, Frad does not consistently benefit from deeper architectures.

Supplementary Table 11. Original runs and hyperparameter sweeps for Frad with different layers. Best results are marked in **bold**.

Layer	6	8	10	12
lr	4e-4	4e-4	4e-4/5e-4/4e-4/1e-4/5e-5/1e-5	4e-4/5e-4/4e-4/1e-4/5e-5/1e-5
homo	**16.0**	**15.2**	**17.0**/19.7/18.9/18.2/20.2/28.0	19.5/**18.1**/20.2/18.7/21.7/26.4
lumo	**14.1**	**14.3**	15.2/17.5/**14.4**/15.2/19.1/25.0	**14.0**/14.5/16.4/18.6/16.9/23.0
gap	**29.2**	**29.8**	33.2/33.5/31.1/**31.0**/35.5/47.9	35.4/38.0/**30.1**/34.3/40.4/45.1

Supplementary Note 8. Raw Results

Supplementary Note 8.1. Results for LBA task

Raw results for the Ligand Binding Affinity prediction (LBA) task are provided in Supplementary Table 12, where the baseline results are derived from ProtNet [33] and ProFSA [8].

Supplementary Note 8.2. Results for MSP task

Raw results for the Mutation Stability Prediction (MSP) task are provided in Supplementary Table 13, where the baseline results are derived from GVP [12] and SiamDiff [39].

Supplementary Note 8.3. Results for Virtual Screening

Raw results for the Virtual Screening task are provided in Supplementary Table 14, where the baseline results are derived from Glide [6], ESM [19] and Uni-Mol [40].

Supplementary Note 9. Memory Efficiency

Supplementary Note 9.1. Efficient Attention Mechanism

Consider the attention mechanism introduced in Eq. (9-12), the query, key matrix $\mathbf{Q}_s, \mathbf{K}_s$ and the concatenated value matrix $\mathbf{V}_s$ share the shape of $\mathbb{R}^{B\times N_{\max}\times S\times 4h_s}$, where $B, N_{\max}, S, h_s$ denote the

Supplementary Table 12. The mean and standard deviations of 3 runs on the LBA dataset. The best results are in **bold** and the second best are underlined.

Model	Sequence Identity 30%			Sequence Identity 60%		
	RMSE↓	Pearson↑	Spearman↑	RMSE↓	Pearson↑	Spearman↑
DeepDTA	1.866 ± 0.080	0.472 ± 0.022	0.471 ± 0.024	1.762 ± 0.261	0.666 ± 0.012	0.663 ± 0.015
B & B	1.985 ± 0.006	0.165 ± 0.006	0.152 ± 0.024	1.891 ± 0.004	0.249 ± 0.006	0.275 ± 0.008
TAPE	1.890 ± 0.035	0.338 ± 0.044	0.286 ± 0.124	1.633 ± 0.016	0.568 ± 0.033	0.571 ± 0.021
ProtTrans	1.544 ± 0.015	0.438 ± 0.053	0.434 ± 0.058	1.641 ± 0.016	0.595 ± 0.014	0.588 ± 0.009
MaSIF	1.484 ± 0.018	0.467 ± 0.020	0.455 ± 0.014	1.426 ± 0.017	0.709 ± 0.008	0.701 ± 0.001
IEConv	1.554 ± 0.016	0.414 ± 0.053	0.428 ± 0.032	1.473 ± 0.024	0.667 ± 0.011	0.675 ± 0.019
Holoprot-Full Surface	1.464 ± 0.006	0.509 ± 0.002	0.500 ± 0.005	1.365 ± 0.038	0.749 ± 0.014	0.742 ± 0.011
Holoprot-Superpixel	1.491 ± 0.004	0.491 ± 0.014	0.482 ± 0.032	1.416 ± 0.022	0.724 ± 0.011	0.715 ± 0.006
ProtNet-Amino Acid	1.455 ± 0.009	0.536 ± 0.012	0.526 ± 0.012	1.397 ± 0.018	0.741 ± 0.008	0.734 ± 0.009
ProtNet-Backbone	1.458 ± 0.003	0.546 ± 0.007	0.550 ± 0.008	1.349 ± 0.019	0.764 ± 0.006	0.759 ± 0.001
ProtNet-All-Atom	1.463 ± 0.001	0.551 ± 0.005	0.551 ± 0.008	1.343 ± 0.025	0.765 ± 0.009	0.761 ± 0.003
Atom3D-3DCNN	1.416 ± 0.021	0.550 ± 0.021	0.553 ± 0.009	1.621 ± 0.025	0.608 ± 0.020	0.615 ± 0.028
Atom3D-ENN	1.568 ± 0.012	0.389 ± 0.024	0.408 ± 0.021	1.620 ± 0.049	0.623 ± 0.015	0.633 ± 0.021
Atom3D-GNN	1.601 ± 0.048	0.545 ± 0.027	0.533 ± 0.033	1.408 ± 0.069	0.743 ± 0.022	0.743 ± 0.027
EGNN-PLM	1.403 ± 0.010	0.565 ± 0.020	0.544 ± 0.010	1.559 ± 0.020	0.644 ± 0.020	0.646 ± 0.020
Uni-Mol	1.520 ± 0.030	0.558 ± 0.000	0.540 ± 0.000	1.619 ± 0.040	0.645 ± 0.020	0.653 ± 0.020
ProFSA	1.377 ± 0.010	$\underline{0.628 \pm 0.010}$	$\underline{0.620 \pm 0.010}$	1.377 ± 0.010	0.764 ± 0.000	0.762 ± 0.010
EPT-Scratch	1.378 ± 0.026	0.604 ± 0.016	0.594 ± 0.017	1.277 ± 0.020	0.787 ± 0.008	0.785 ± 0.008
EPT-Molecule	1.336 ± 0.030	0.621 ± 0.016	0.602 ± 0.017	1.243 ± 0.022	0.802 ± 0.008	$\underline{0.800 \pm 0.009}$
EPT-Protein	$\underline{1.329 \pm 0.013}$	$\underline{0.628 \pm 0.004}$	0.613 ± 0.004	$\underline{1.235 \pm 0.016}$	$\underline{0.804 \pm 0.006}$	$\underline{0.800 \pm 0.005}$
EPT-MultiDomain	$\mathbf{1.322 \pm 0.025}$	$\mathbf{0.644 \pm 0.016}$	$\mathbf{0.630 \pm 0.014}$	$\mathbf{1.227 \pm 0.013}$	$\mathbf{0.811 \pm 0.003}$	$\mathbf{0.803 \pm 0.004}$

batch size, the maximum number of atoms, the number of heads and the size of each head's hidden state. Such shape consistency enables the usage of previous memory efficient techniques [17] that provide the interface of attention biases for **D** and **R**. We provide the pseudo codes in PyTorch [26] style as follows.

Supplementary Table 13. Mean and standard deviation across 3 runs on mutation stability prediction (MSP). The best scores are marked in **bold** and the second best <u>underlined</u>.

	Model	AUROC
w/o Pretrain	GCN	0.621 ± 0.009
	Atom3D-CNN	0.574 ± 0.005
	Atom3D-ENN	0.574 ± 0.040
	GVP	<u>0.680 ± 0.015</u>
	GearNet-Edge	0.633 ± 0.067
	EPT (ours)	$\mathbf{0.705 \pm 0.025}$
w/ Pretrain	Multiview Contrast	0.646 ± 0.006
	DiffPreT	0.680 ± 0.018
	SiamDiff	0.698 ± 0.020
	EPT (ours)	$\mathbf{0.720 \pm 0.008}$

Supplementary Table 14. Results on 3 splits of Docked PDBBind.

Split	Model	|Pearson|↑	|Spearman|↑	Top-1 Acc↑	AvgRank↓
id30	Glide	0.232	0.239	0.460	0.251 ± 0.228
	ESM+Uni-Mol	0.201	0.228	0.202	0.371 ± 0.248
	EPT	0.422	0.430	0.454	0.249 ± 0.215
id60	Glide	0.232	0.239	0.460	0.251 ± 0.228
	ESM+Uni-Mol	0.180	0.163	0.236	0.347 ± 0.249
	EPT	0.445	0.451	0.454	0.258 ± 0.216
scaffold	Glide	0.232	0.239	0.460	0.251 ± 0.228
	ESM+Uni-Mol	0.247	0.256	0.230	0.338 ± 0.230
	EPT	0.506	0.510	0.470	0.238 ± 0.206

```
from xformers.ops import memory_efficient_attention

def equivariant_memory_efficient_self_attention(H_in, V_in, D, R, mask):

    '''
    Params:
        H_in: B * N_max * h
        V_in: B * N_max * 3 * h
        D: B * N_max * N_max
        R: B * N_max * N_max
        mask: B * N_max
    Returns:
        H_out: B * N_max * h
        V_out: B * N_max * 3 * h
    '''
    # Eq. (6)
    Query_s = linear_scalar_Q(H_in).view(B, N_max, S, h_s * 4)
    Key_s = linear_scalar_K(H_in).view(B, N_max, S, h_s * 4)
    Value_s_scalar = linear_scalar_K(H_in).view(B, N_max, S, h_s)
    Value_s_vector = linear_scalar_K(V_in).view(B, N_max, 3, S, h_s)
    # B * N_max * S * 3h_s
    Value_s_vector = Value_s_vector.transpose(2,3).flatten(start_dim=-2)
    # B * N_max * S * 4h_s
```

```
    Value_s = cat([Value_s_scalar, Value_s_vector], dim=-1)
    # Eq. (7)
    bias = R - D
    bias = bias.masked_fill(mask.unsqueeze(1).unsqueeze(2) == 0, float("inf"))
    bias = bias.expand(-1, S, -1, -1)
    HV = memory_efficient_attention(
        query = Query_s,
        key = Key_s,
        value = Value_s,
        attn_bias = bias
    )
    H_s = HV[..., :h_s]
    V_s = HV[..., h_s:].view(B, N_max, S, 3, h_s).transpose(2,3)
    # Eq. (9)
    H_out = linear_scalar_O(H_s.view(B, N_max, h))
    V_out = linear_vector_O(V_s.view(B, N_max, 3, h))
    return H_out, V_out
```

Supplementary Note 9.2. Comparison on Transformer-based Backbones

We further compared the GPU memory consumption of our model, a 6-layer, 512-hidden EPT, with two previous Transformer-based backbones: the 6-layer, 512-hidden TorchMD-Net [29] and the 6-layer, 128-hidden, 3-degree Equiformer [18], which have 31M, 19M, and 18M parameters, respectively. Our tests measured memory usage of one forward step on point clouds with 32 to 2048 nodes, sampled uniformly within a sphere of radius $\sqrt[3]{N}$, and connected by edges within a 4.0 cutoff distance. Supplementary Fig. 6 illustrates that EPT is consistently more memory-efficient across various node counts. This efficiency enables our model to effectively process large-scale point clouds, facilitating the study of expansive molecular systems.

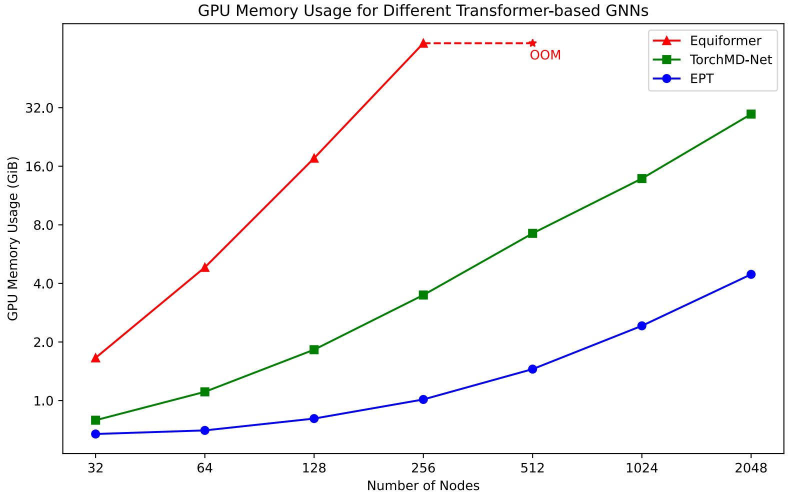

Supplementary Fig. 6 | **GPU Memory Usage for Transformer-based GNNs.** GPU memory usage comparison of three Transformer-based backbones as the number of nodes increases. EPT demonstrates a more memory-efficient scaling behavior compared to TorchMD and EquiFormer. EquiFormer encounters an Out-of-Memory (OOM) error at 512 nodes on a NVIDIA Tesla A800 with 80G GPU memory.

Supplementary Note 10. Impact of Decoy Numbers

To benchmark the virtual screening task, we re-docked 10 decoys for each complex to serve as negative candidates and required the model to identify the true ligand among all samples. In real-world scenarios, virtual screening typically involves ranking potential binders from larger compound libraries. However, generating millions of docked samples for each binding site is computationally prohibitive. To further investigate the effect of the number of negative samples, we re-docked 50 more decoys for each complex, finetuned EPT on the id30 split with the same setting of Supplementary Table 8, and evaluated the model's ranking performance across varying numbers of decoys on the test sets. The results in Fig. 7 show that the evaluation metric remains stable regardless of whether 10 or more decoys are used per target. Therefore, we adopt 10 decoys as the default setting in our experiments.

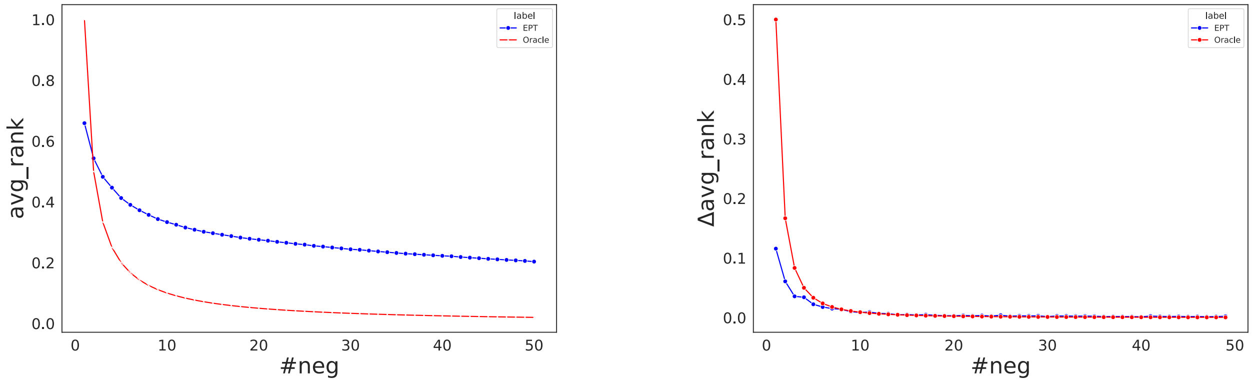

Supplementary Fig. 7 | The effect of the number of decoys on the average rank metric during inference. avg_rank(n) and Δavg_rank(n) = avg_rank(n) − avg_rank($n+1$) over the number of negative samples n on the id30 split. The Oracle method indicates positive samples are always ranked as top-1.

Supplementary Note 11. Discussion on Unified Modelling for Atomic Systems

Recent advances in atomic representation learning have given rise to a variety of unified modeling paradigms that aim to bridge domains and tasks, which can be broadly grouped into three complementary directions, including structure-aware generalist models, unified 3D generative frameworks, and sequence-level foundation models. Structure-aware generalist models are designed to learn transferable 3D representations across domains. Uni-Mol [40] exemplifies this approach with a dual-tower pretraining strategy that separately encodes small molecules and protein pockets before alignment on binding tasks. BIT [41] introduces the concept of Mixture-of-Domain-Experts (MoDE), utilizing distinct FFN layers to model small molecules and proteins separately. EPT belongs to this family, and distinguishes itself by emphasizing biomolecular systems, adopting an all-atom unified representation, and leveraging block-level denoising to capture hierarchical structural features.

A second line of research focuses on 3D generative frameworks, where the objective is to directly synthesize molecular structures within a unified generative model such as diffusion or flow matching. The All-atom Diffusion Transformer (ADiT) [13] demonstrates that molecules and periodic crystals

can be generated jointly by leveraging a shared latent diffusion model. AlphaFold3 [2] extends generative modeling to proteins, RNAs, and complexes, unifying structural prediction across biomolecular modalities. PharMolixFM [22] builds a multimodal generative foundation at the all-atom level, enabling interaction modeling across diverse biochemical systems. Unlike these generative works, EPT focuses on representation learning and predictive understanding, but its unified and equivariant architecture provides a promising backbone for future generative frameworks.

A third emerging direction is sequence-level foundation models, which treat molecular modalities as symbolic sequences and leverage language-modeling paradigms for cross-domain generalization. NatureLM [34] unifies chemical and biological sequences in a natural-language-style pretraining framework. UniGenX [38] extends this principle by incorporating both sequence and structure in its generation. Uni-3DAR [20] leverages autoregressive tokenization over octree-based spatial tokens derived from 3D structure to unify both generation and understanding of 3D structure in a sequence-level framework. These approaches are orthogonal to EPT since they operate purely on symbolic sequences rather than 3D geometries. However, there exist future directions to combine these two perspectives. Sequence-level generalists could provide coarse-grained priors that are refined by EPT's structure-aware modeling, leading to multi-scale unification across symbolic and geometric representations.

Supplementary References

1. M. Abraham, A. Alekseenko, V. Basov, C. Bergh, E. Briand, A. Brown, M. Doijade, G. Fiorin, S. Fleischmann, S. Gorelov, G. Gouaillardet, A. Grey, M. E. Irrgang, F. Jalalypour, J. Jordan, C. Kutzner, J. A. Lemkul, M. Lundborg, P. Merz, and E. Lindahl. *GROMACS 2024.2 Manual (2024.2)*. Zenodo, https://doi.org/10.5281/zenodo.11148638, 2024.

2. Josh Abramson, Jonas Adler, Jack Dunger, Richard Evans, Tim Green, Alexander Pritzel, Olaf Ronneberger, Lindsay Willmore, Andrew J Ballard, Joshua Bambrick, et al. Accurate structure prediction of biomolecular interactions with alphafold 3. *Nature*, 630(8016):493–500, 2024.

3. Giovanni Bussi, Davide Donadio, and Michele Parrinello. Canonical sampling through velocity rescaling. *The Journal of chemical physics*, 126(1), 2007.

4. David A Case, H Metin Aktulga, Kellon Belfon, Ido Ben-Shalom, Scott R Brozell, David S Cerutti, Thomas E Cheatham III, Vinícius Wilian D Cruzeiro, Tom A Darden, Robert E Duke, et al. *Amber 2021*. University of California, San Francisco, 2021.

5. Shikun Feng, Yuyan Ni, Yanyan Lan, Zhi-Ming Ma, and Wei-Ying Ma. Fractional denoising for 3d molecular pre-training. In *International Conference on Machine Learning*, pages 9938–9961. PMLR, 2023.

6. Richard A Friesner, Jay L Banks, Robert B Murphy, Thomas A Halgren, Jasna J Klicic, Daniel T Mainz, Matthew P Repasky, Eric H Knoll, Mee Shelley, Jason K Perry, et al. Glide: a new approach for rapid, accurate docking and scoring. 1. method and assessment of docking accuracy. *Journal of medicinal chemistry*, 47(7):1739–1749, 2004.

7. M. J. Frisch, G. W. Trucks, H. B. Schlegel, G. E. Scuseria, M. A. Robb, J. R. Cheeseman, G. Scalmani, V. Barone, G. A. Petersson, H. Nakatsuji, X. Li, M. Caricato, A. V. Marenich,

J. Bloino, B. G. Janesko, R. Gomperts, B. Mennucci, H. P. Hratchian, J. V. Ortiz, A. F. Izmaylov, J. L. Sonnenberg, F. Williams, Ding, F. Lipparini, F. Egidi, J. Goings, B. Peng, A. Petrone, T. Henderson, D. Ranasinghe, V. G. Zakrzewski, J. Gao, N. Rega, G. Zheng, W. Liang, M. Hada, M. Ehara, K. Toyota, R. Fukuda, J. Hasegawa, M. Ishida, T. Nakajima, Y. Honda, O. Kitao, H. Nakai, T. Vreven, K. Throssell, J. A. Montgomery, J. E. Peralta, F. Ogliaro, M. J. Bearpark, J. J. Heyd, E. N. Brothers, K. N. Kudin, V. N. Staroverov, T. A. Keith, R. Kobayashi, J. Normand, K. Raghavachari, A. P. Rendell, J. C. Burant, S. S. Iyengar, J. Tomasi, M. Cossi, J. M. Millam, M. Klene, C. Adamo, R. Cammi, J. W. Ochterski, R. L. Martin, K. Morokuma, O. Farkas, J. B. Foresman, and D. J. Fox. *Gaussian 09 Rev. D.01*. Gaussian Inc., Wallingford, 2016.

8. Bowen Gao, Yinjun Jia, Yuanle Mo, Yuyan Ni, Weiying Ma, Zhiming Ma, and Yanyan Lan. Self-supervised pocket pretraining via protein fragment-surroundings alignment. *arXiv preprint arXiv:2310.07229*, 2023.

9. Jonathan Godwin, Michael Schaarschmidt, Alexander L Gaunt, Alvaro Sanchez-Gonzalez, Yulia Rubanova, Petar Veličković, James Kirkpatrick, and Peter Battaglia. Simple gnn regularisation for 3d molecular property prediction and beyond. In *International Conference on Learning Representations*, 2021.

10. Rui Jiao, Jiaqi Han, Wenbing Huang, Yu Rong, and Yang Liu. Energy-motivated equivariant pretraining for 3d molecular graphs. In *Proceedings of the AAAI Conference on Artificial Intelligence*, volume 37, pages 8096–8104, 2023.

11. Wengong Jin, Siranush Sarkizova, Xun Chen, Nir Hacohen, and Caroline Uhler. Unsupervised protein-ligand binding energy prediction via neural euler's rotation equation. *arXiv preprint arXiv:2301.10814*, 2023.

12. Bowen Jing, Stephan Eismann, Pratham N Soni, and Ron O Dror. Equivariant graph neural networks for 3d macromolecular structure. *arXiv preprint arXiv:2106.03843*, 2021.

13. Chaitanya K Joshi, Xiang Fu, Yi-Lun Liao, Vahe Gharakhanyan, Benjamin Kurt Miller, Anuroop Sriram, and Zachary W Ulissi. All-atom diffusion transformers: Unified generative modelling of molecules and materials. *arXiv preprint arXiv:2503.03965*, 2025.

14. Masaaki Kawata and Umpei Nagashima. Particle mesh ewald method for three-dimensional systems with two-dimensional periodicity. *Chemical Physics Letters*, 340(1-2):165–172, 2001.

15. Edward King, Erick Aitchison, Han Li, and Ray Luo. Recent developments in free energy calculations for drug discovery. *Frontiers in Molecular Biosciences*, 8:712085, 2021.

16. Adam Leach, Sebastian M Schmon, Matteo T. Degiacomi, and Chris G. Willcocks. Denoising diffusion probabilistic models on SO(3) for rotational alignment. In *ICLR 2022 Workshop on Geometrical and Topological Representation Learning*, 2022.

17. Benjamin Lefaudeux, Francisco Massa, Diana Liskovich, Wenhan Xiong, Vittorio Caggiano, Sean Naren, Min Xu, Jieru Hu, Marta Tintore, Susan Zhang, Patrick Labatut, and Daniel Haziza. xformers: A modular and hackable transformer modelling library. https://github.com/facebookresearch/xformers, 2022.

18. Yi-Lun Liao and Tess Smidt. Equiformer: Equivariant graph attention transformer for 3d atomistic graphs. *arXiv preprint arXiv:2206.11990*, 2022.

19. Zeming Lin, Halil Akin, Roshan Rao, Brian Hie, Zhongkai Zhu, Wenting Lu, Nikita Smetanin, Allan dos Santos Costa, Maryam Fazel-Zarandi, Tom Sercu, Sal Candido, et al. Language models of protein sequences at the scale of evolution enable accurate structure prediction. *bioRxiv*, 2022.

20. Tian Lu. A comprehensive electron wavefunction analysis toolbox for chemists, multiwfn. *The Journal of Chemical Physics*, 161(8), 2024.

21. Tian Lu. Sobtop (version 1.0), 2024. Accessed: June 18, 2024.

22. Yizhen Luo, Jiashuo Wang, Siqi Fan, and Zaiqing Nie. Pharmolixfm: All-atom foundation models for molecular modeling and generation. *arXiv preprint arXiv:2503.21788*, 2025.

23. Chunlong Ma, Michael Dominic Sacco, Brett Hurst, Julia Alma Townsend, Yanmei Hu, Tommy Szeto, Xiujun Zhang, Bart Tarbet, Michael Thomas Marty, Yu Chen, et al. Boceprevir, gc-376, and calpain inhibitors ii, xii inhibit sars-cov-2 viral replication by targeting the viral main protease. *Cell research*, 30(8):678–692, 2020.

24. James A Maier, Carmenza Martinez, Koushik Kasavajhala, Lauren Wickstrom, Kevin E Hauser, and Carlos Simmerling. ff14sb: improving the accuracy of protein side chain and backbone parameters from ff99sb. *Journal of chemical theory and computation*, 11(8):3696–3713, 2015.

25. Michele Parrinello and Aneesur Rahman. Polymorphic transitions in single crystals: A new molecular dynamics method. *Journal of Applied physics*, 52(12):7182–7190, 1981.

26. Adam Paszke, Sam Gross, Francisco Massa, Adam Lerer, James Bradbury, Gregory Chanan, Trevor Killeen, Zeming Lin, Natalia Gimelshein, Luca Antiga, et al. Pytorch: An imperative style, high-performance deep learning library. *Advances in neural information processing systems*, 32, 2019.

27. Yang Song and Stefano Ermon. Generative modeling by estimating gradients of the data distribution. *Advances in neural information processing systems*, 32, 2019.

28. Davison E Soper. *Classical field theory.* Courier Dover Publications, 2008.

29. Philipp Thölke and Gianni De Fabritiis. Torchmd-net: equivariant transformers for neural network based molecular potentials. *arXiv preprint arXiv:2202.02541*, 2022.

30. Raphael JL Townshend, Martin Vögele, Patricia Suriana, Alexander Derry, Alexander Powers, Yianni Laloudakis, Sidhika Balachandar, Bowen Jing, Brandon Anderson, Stephan Eismann, et al. Atom3d: Tasks on molecules in three dimensions. *arXiv preprint arXiv:2012.04035*, 2020.

31. Mario S Valdés-Tresanco, Mario E Valdés-Tresanco, Pedro A Valiente, and Ernesto Moreno. gmx_mmpbsa: a new tool to perform end-state free energy calculations with gromacs. *Journal of chemical theory and computation*, 17(10):6281–6291, 2021.

32. Junmei Wang, Romain M Wolf, James W Caldwell, Peter A Kollman, and David A Case. Development and testing of a general amber force field. *Journal of computational chemistry*, 25(9):1157–1174, 2004.

33. Limei Wang, Haoran Liu, Yi Liu, Jerry Kurtin, and Shuiwang Ji. Learning hierarchical protein representations via complete 3d graph networks. In *International Conference on Learning Representations (ICLR)*, 2023.

34. Yingce Xia, Peiran Jin, Shufang Xie, Liang He, Chuan Cao, Renqian Luo, Guoqing Liu, Yue Wang, Zequn Liu, Yuan-Jyue Chen, et al. Naturelm: Deciphering the language of nature for scientific discovery. *arXiv e-prints*, pages arXiv–2502, 2025.

35. Minkai Xu, Lantao Yu, Yang Song, Chence Shi, Stefano Ermon, and Jian Tang. Geodiff: A geometric diffusion model for molecular conformation generation. *arXiv preprint arXiv:2203.02923*, 2022.

36. Muhammad Yasir, Jinyoung Park, Eun-Taek Han, Jin-Hee Han, Won Sun Park, and Wanjoo Chun. Investigating the inhibitory potential of flavonoids against aldose reductase: insights from molecular docking, dynamics simulations, and gmx_mmpbsa analysis. *Current Issues in Molecular Biology*, 46(10):11503–11518, 2024.

37. Sheheryar Zaidi, Michael Schaarschmidt, James Martens, Hyunjik Kim, Yee Whye Teh, Alvaro Sanchez-Gonzalez, Peter Battaglia, Razvan Pascanu, and Jonathan Godwin. Pre-training via denoising for molecular property prediction. *arXiv preprint arXiv:2206.00133*, 2022.

38. Gongbo Zhang, Yanting Li, Renqian Luo, Pipi Hu, Zeru Zhao, Lingbo Li, Guoqing Liu, Zun Wang, Ran Bi, Kaiyuan Gao, et al. Unigenx: Unified generation of sequence and structure with autoregressive diffusion. *arXiv preprint arXiv:2503.06687*, 2025.

39. Zuobai Zhang, Minghao Xu, Aurelie Lozano, Vijil Chenthamarakshan, Payel Das, and Jian Tang. Pre-training protein encoder via siamese sequence-structure diffusion trajectory prediction. In *Annual Conference on Neural Information Processing Systems*, 2023.

40. Gengmo Zhou, Zhifeng Gao, Qiankun Ding, Hang Zheng, Hongteng Xu, Zhewei Wei, Linfeng Zhang, and Guolin Ke. Uni-mol: a universal 3d molecular representation learning framework, 2023.

41. Yiheng Zhu, Mingyang Li, Junlong Liu, Kun Fu, Jiansheng Wu, Qiuyi Li, Mingze Yin, Jieping Ye, Jian Wu, and Zheng Wang. A generalist cross-domain molecular learning framework for structure-based drug discovery. *arXiv preprint arXiv:2503.04362*, 2025.
